# The unmet needs of childhood cancer survivors in long‐term follow‐up care: A qualitative study

**DOI:** 10.1002/pon.5593

**Published:** 2020-12-18

**Authors:** Manya Jerina Hendriks, Erika Harju, Gisela Michel

**Affiliations:** ^1^ Department of Health Sciences and Medicine University of Lucerne Lucerne Switzerland; ^2^ Clinical Ethics University Hospital Zurich University of Zurich Zurich Switzerland

**Keywords:** aftercare, cancer survivors, health services needs and demand, long‐term cancer survivors, neoplasm, needs assessment, patient preference, psycho‐oncology, psychosocial support systems, qualitative research

## Abstract

**Objective:**

Evidence suggests benefits of long‐term follow‐up care attendance for childhood cancer survivors, but studies show poor inclusion of survivorship issues and needs. While information needs of childhood cancer survivors have been addressed previously, few studies specifically investigated the supportive care needs of survivors beyond the domain of information and communication. Therefore, this qualitative study aimed to assess the unmet needs of childhood cancer survivors with regards to their long‐term survivorship.

**Methods:**

Childhood Cancer Switzerland invited survivors of childhood cancer to participate in our study. We used semistructured interviews to assess survivors' experiences regarding the impact of their disease and the (un)met needs during their survivorship. Data analysis followed the principles of qualitative content analysis.

**Results:**

Interviews were conducted with 28 childhood cancer survivors (mean age 31 years, age at diagnosis 9 years, time since end of treatment 19 years). Key themes in relation to unmet needs of survivors were (1) lacking psychosocial support, (2) lacking collaboration and decentralization of care, (3) starting from zero, and (4) need for centralized, specialized, and individualized services.

**Conclusions:**

Our findings revealed a demand for integrating psychosocial support in long‐term follow‐up care and a strong need for personalized, centralized, and interdisciplinary long‐term follow‐up care. Current established interdisciplinary clinics should be further extended to provide centralized, personalized, and evidence‐based long‐term follow‐up care including adequate psychosocial support for all childhood cancer survivors.

## 1 BACKGROUND

Long‐term follow‐up care of childhood cancer survivors has received increasing attention since treatment improvements have led to a growing number of survivors over the past decades.^1^ Currently, more than 80% of children diagnosed with cancer survive and ten‐year survival has significantly improved (>87%).^2^ However, with successful treatment, a life‐long risk of adverse health effects for childhood cancer survivors arises^3^ including medical and psychosocial issues.^4^, ^5^, ^6^ To improve quality of life and diagnose treatable late effects early, providing high quality long‐term follow‐up care is of imminent importance. In fact, the goal of a timely long‐term follow‐up care is to prevent, detect, or decrease severity of late treatment effects through preventative therapeutic measures, health promotion, and psychosocial support.^7^ Recently, the need to implement effective and efficient long‐term follow‐up models of care has become recognized in several countries.^8^

In Switzerland, follow‐up care has long been available in the first 5–10 years after end of treatment and most centers provide transition from pediatric to adult care (usually at 18–20 years). That is to say general practitioners or clinical oncologist provide adult childhood cancer survivors care, albeit mostly without detailed evidence‐based information and instruction on long‐term follow‐up. Once transitioned into adult care, long‐term follow‐up is poorly standardized.^9^, ^10^, ^11^ Currently, only four centers provide interdisciplinary long‐term follow‐up care with pediatric oncology for adult childhood cancer survivors. One of these centers focuses on childhood cancer survivors previously lost to long‐term follow‐up after transitioning into adult care.^12^ More recently, some clinics have started to provide detailed survivorship care plans including treatment summaries as recommended by guidelines.^9^ ^,^ ^12^ However, childhood cancer survivors and health care professionals (HCPs) in Switzerland and other countries continue to describe lacking long‐term follow‐up programs and report heterogeneity in aftercare among centers.^8^ ^,^ ^13^ ^,^ ^14^ Evidence has shown poor inclusion of survivorship issues and needs.^15^, ^16^, ^17^, ^18^ It is important to address unmet needs as studies have shown that survivors with information needs experience more psychological distress and lower quality of life.^19^, ^20^, ^21^ Especially, unmet information needs are known to negatively impact long‐term follow‐up attendance or seeking of medical care.^10^ ^,^ ^19^, ^20^, ^21^ While there is some evidence on childhood cancer survivors' information needs,^19^ ^,^ ^22^ few studies specifically investigated the supportive care needs beyond the domain of information and communication.^23^ ^,^ ^24^ However, childhood cancer survivors may experience other types of unmet needs. Recently, we have shown that alongside largely unmet informational needs, a subset of Swiss childhood cancer survivors further report unmet psychosocial needs.^25^ In this study, we build on these quantitative results with an in‐depth understanding of the unmet needs of childhood cancer survivors.

With the increasing number of childhood cancer survivors, optimizing long‐term follow‐up care is essential to provide efficient and effective, high‐quality care for all survivors.^26^ ^,^ ^27^ In turn, addressing childhood cancer survivors' needs might help increase their long‐term follow‐up attendance.^10^ ^,^ ^24^ ^,^ ^27^ Thus, we aimed to assess the supportive care needs of childhood cancer survivors with regards to their long‐term survivorship.

## 2 METHODS

This paper builds on the results of a cross‐sectional survey on the impact of cancer and childhood cancer survivors' unmet needs during survivorship. For the current study, we used a qualitative research design^28^ and conducted semistructured interviews with a subset of participants from the survey. The Ethics Committee Northwest and Central Switzerland (Study‐ID: EKNZ 2017‐01758) approved the study.

### 2.1 Participants and procedure

Childhood cancer survivors had been identified through Childhood Cancer Switzerland, the umbrella organization of childhood cancer associations in pediatric oncology in Switzerland.^25^ Participants were eligible if they were aged ≥ 18 years at time of study, diagnosed with cancer ≤ 18 years of age, ended treatment ≥ 2 years before study, were Swiss residents, and spoke German or English.

Childhood Cancer Switzerland sent an e‐mail invitation to all its registered survivors (*n* = 132) to participate in a cross‐sectional survey. We sent a reminder to nonresponders after 2 months. Additional participants were invited through an open electronic link that was circulated among Swiss survivors' networks on social platforms (e.g., Twitter, survivors' WhatsApp groups) and in survivor meetings. Out of 132 eligible participants, 63 survivors returned the questionnaire and an additional six childhood cancer survivors were reached through social media platforms resulting in 69 participants. These survey participants were invited to take part in an interview in order to obtain a more nuanced understanding of their experiences, preferences, and needs regarding their long‐term survivorship.

### 2.2 Data collection

Survey participants who were interested in the qualitative study shared their contact information with the study team. Interviews were scheduled at a location selected by childhood cancer survivors (i.e. participants' home, workplace, or a meeting room at a University). The first author conducted all interviews between November 2017 and February 2019. The interviews were conducted until theoretical saturation was achieved.^28^ Before the interviews, participants received written and oral information about the study. They were ensured anonymity and provided written informed consent. The interview guide focused on participants' experiences regarding the impact of their disease and (un)met needs during their survivorship (see Supplemental Appendix). The interviews were audio recorded (length: 39–117 min, approximately 89 min) and transcribed verbatim.

### 2.3 Data analysis

Data analysis followed the principles of qualitative content analysis, which takes into account previously defined research questions outlined by the literature and allows categories to emerge out of the data. Two members of the research team (MJH and EH) reviewed the transcripts to identify emerging themes and together developed an initial coding frame utilizing a deductive‐inductive procedure.^29^ This approach integrates elements from grounded theory such as theoretical memos and iteration to generate descriptive results and conceptual models of the topics under study.^29^ Hence, this approach is focused on the importance of context in determining meaning, that is data driven and iterative. An initial coding scheme was developed based on our research question, interview guide, and reviewed literature. First, preliminary codes were generated through systematic coding of the data by MJH and EH. Second, identified codes were reviewed and refined. Consensus for coding was reached. All transcripts were recoded at the end of the process using the finalized coding structure.^29^ Qualitative data organization and aggregation were facilitated by Atlas.ti 8.3 (data available upon written request).

## 3 RESULTS

### 3.1 Sample characteristics

Out of 61 interested survivors, 28 (46%) participated in an interview. The most frequently reported diagnosis was leukemia (10/28), and mean time since end of treatment was 19 years (Table 1). The key themes that emerged in relation to the unmet needs of childhood cancer survivors were (1) lacking psychosocial support, (2) lacking collaboration and decentralization of care, (3) starting from zero, and (4) need for centralized, specialized, and individualized services.

**TABLE 1:** Participants' characteristics

Total	*N* = 28 (100%)
Gender	
Male	9 (32.1)
Female	19 (67.9)
Age at study	
≤25 years	11 (39.3)
26–30 years	5 (17.9)
31–35 years	4 (14.3)
>35 years	8 (28.6)
Nationality^a^	
Swiss	24 (85.7)
Swiss and other	4 (14.3)
In a relationship	
Yes	7 (25.0)
No	21 (75.0)
Children	
Yes	6 (21.4)
No	22 (78.6)
Education	
Compulsory schooling	4 (14.3)
Vocational training	13 (46.4)
Upper secondary	6 (21.4)
University degree	5 (17.9)
Employment	
Yes	24 (85.7)
No, in education	3 (10.7)
No, homemaker	1 (3.6)
Diagnosis	
Leukemia	10 (35.7)
Lymphoma	5 (17.9)
Central nervous system tumor	3 (10.7)
Renal tumor	3 (10.7)
Bone tumor	5 (17.9)
Soft tissue sarcoma	2 (7.1)
Age at diagnosis (years)	
0–5	7 (25.0)
6–11	10 (35.7)
12–18	11 (39.3)
Treatment	
Surgery only	1 (3.6)
Chemotherapy^b^	14 (50.0)
Radiation^c^	10 (35.7)
Stem cell transplantation	3 (10.7)
Time since end of treatment (years)	
≤ 5	3 (10.7)
6–15	9 (32.1)
16–25	6 (21.4)
> 25	10 (35.7)
Late effects	
Yes	18 (64.3)
No	10 (35.7)
Type of late effects^d^	
Physical	12 (75.0)
Physical and psychological	4 (25.0)
Follow‐up attendance	
Yes	15 (53.6)
No, completed	13 (46.4)
Relapse	
Yes	7 (25.0)
No	21 (75.0)
Second malignancy	
Yes	6 (21.4)
No	22 (78.6)

Years	Mean (range)
Age at study	31.4 (18–55)
Age at diagnosis	9.3 (0.5–16)
Time since end of treatment	19.1 (2–38)

^a^Other nationalities: German, Dutch, Italian.

^b^May have included surgery, but no radiation.

^c^May have included surgery and/or chemotherapy.

^d^Missings.

### 3.2 Lacking psychosocial support

Regardless of time since end of treatment most childhood cancer survivors described how provision of psychological or psychosocial support was lacking. This means that psychological and social support was lacking in follow‐up as well as in long‐term follow‐up. Childhood cancer survivors mentioned that it was difficult to get support from many services and “*there is not really anything*.” Either no point of contact exists or the service is overwhelmed with requests. One childhood cancer survivor described how HCPs would merely ask whether he was “*doing ok*” with a focus on his physical wellbeing while offering “*zero psychological* *support*.” In addition, this burden is further exacerbated when there is limited information or faulty communication channels for finding formal support. Survivors reported feelings of “*being lost*.” One survivor described how during her appointment, even her HCP could not provide further information on where to find psychological support.

*I had a checkup, one of these regular checkups in the hospital and there I mentioned possible psychological support. […] At some point I was told, no, I had to look externally. Okay, good. Thanks. […] I'm somewhat lost myself, where do I go to ask now, because, I don't know either with this follow‐up care. I asked the doctors. I mean, if the doctors can't really tell you, well then, where do you go to ask? Then you are still left with nothing*. – Female survivor, 6–15 years since end of treatment, no late effects (Interview #26F)

Similarly, other childhood cancer survivors described lacking psychosocial support and needed to “*fend for oneself*” after treatment. A majority of survivors described the long and difficult process of searching for psychosocial support or claiming assistance from specific institutions. This encompassed arranging extra tutoring in school, leave of absence for medical appointments during school or work time, advice on financial or insurance matters, or finding a psychologist with knowledge on childhood cancer.

### 3.3 Lacking collaboration and decentralization of care

Furthermore, many childhood cancer survivors described a lack of interdisciplinary collaboration amongst HCPs. They reported having several different HCPs from different disciplines that they visit monthly or yearly for their long‐term follow‐up care. However, sometimes communication and collaboration between these disciplines was lacking, which caused distress. As one survivor put it; “*it is every department for itself*,” in which quality curtails when everyone is working only within the limitations of their own medical field. Another survivor explained how the lack of collaboration lead to “*disorganized long‐term follow‐up care*” such that in the end she often did not know to whom she could go with questions regarding her care.

Not only did childhood cancer survivors consider long‐term follow‐up as lacking a well‐established structure for collaboration, survivors also mentioned the (dis)stress from decentralized care. Survivors reported having many *different* appointments on *different* days in *different* locations with *different* HCPs. All survivors considered long‐term follow‐up appointments to be a loss of their (economic) productivity and a great organizational effort. One childhood cancer survivor reported having had five to seven appointments in one week. This meant she had to travel to two different hospitals to visit her two specialists. In turn, she missed school, and had to additionally justify her absence.

### 3.4 Starting from zero

Due to the lacking services and decentralization, survivors reported that every survivor needs to “*reinvent the wheel*” in managing care and finding support. This was considered inefficient and burdensome. Often survivors found access to formal support services (e.g. from governmental, charitable or private institutions) by undertaking individual action or through their informal network of support. Most were dependent on their informal network of support (i.e. family members, friends and social networks). For example, childhood cancer survivors had found access to psychosocial, medical or rehabilitation support services through family members, friends or devoted neighbors that gave advice or functioned as gatekeepers and put them in contact with the institutions that offered formal support.

Childhood cancer survivors illustrated how relying on individual action or personal networks holds two risks. First, childhood cancer survivors raised that the extent of individual organization amounted to only “*checking [medical care] when I was feeling bad, when I had problems*” or not attending long‐term follow‐up at all. Second, those without a well‐established informal network of support could experience additional barriers to accessing formal support services. One survivor mentioned how this could be considered “*unfair*.”

### 3.5 Need for centralized, specialized, and individualized services

In light of the above, a majority of childhood cancer survivors called for a central resource or “*point of contact*.” This would unite several services or would provide coherent information on where to find such services (Table 2). Furthermore, they wanted centralized long‐term follow‐up care provided by specialized HCPs with knowledge on survivorship. As such, “*a centralized interdisciplinary”* service was considered *“the first priority”* to improving long‐term follow‐up for survivors.

*Firstly, you can really* save *a lot of survivors' stress, and secondly your medical home‐base has to be warranted somehow. […] this will be a relief regarding appointments. That there is actually one person—or two people—who can bundle their knowledge about a patient*. – Male survivor, ≥ 25 years since end of treatment, late effects (Interview #3M).

**TABLE 2:** Points of contact for long‐term follow‐up care

Types	Quotes
Centralized	It would have really helped to have someone who could figure it out, for whom perhaps it would also not take so much time and effort. Such a person could have really taken care of the situation. But I do not think such a person exists. I Think that is really something to strive for. Really someone expert on childhood cancer specific concerns, so someone can really support you.—Male survivor, ≥ 25 years since end of treatment, late effects (interview #3M)
Psychological	So I think, when you are really acutely ill and I mean, if you already say, I need help, but that you then have to wait 3,4 weeks for an appointment just because you are not suicidal, I think that is quite strange […] Yes, I think it would actually be good if there was like a point of contact, like an emergency phone number, where you can call and that they would just quickly and at any time have an appointment where you can really go, because really, I think it's insane, that you have to wait so long and I mean in this state I couldn't go to work but I knew I wanted to go back to work soon because it would be good for me if I could go to work and then just sit at home for a month and just simply be, well that startled me pretty much I have to say.—Female survivor, 6–15 years since end of treatment, late effects (interview #18F)
Psychosocial	If there really had to be a point of contact, then it would really be for questions like everyday life, education, profession, partnership or even reproduction or […] just nutrition, sports or maybe also, like, just central places with just people who really understand you […] that would be somewhat the most important thing that is needed from a point of contact.—Female survivor, 16–25 years since end of treatment, late effects (interview #21F)

Childhood cancer survivors further distinguished that care should be tailored to survivors' needs. They often referred to individualized long‐term follow‐up care and information. Survivors described how needs for information change with age, along the survivorship trajectory.

In fact, some survivors had attended and others mentioned interest in a quite newly developed centralized and interdisciplinary long‐term follow‐up center for adult survivors of childhood cancer who have been lost to follow‐up after transitioning into adult care. Those that attended were satisfied; one survivor explained that “*this was the first time that all examinations were conducted in one day*” in which he visited various specialists. Long‐term follow‐up care was specifically designed for him and the effects of his cancer treatment. Although these developments were positive and should serve as a standard approach, childhood cancer survivors reiterated that access to long‐term follow‐up care should not remain to be contingent on the individual to take action. They found it important that long‐term follow‐up was communicated and proactively offered to all, also to long‐term (10–20 years since end of treatment) or very long‐term (> 20 years) survivors who were lost to follow‐up.

*But for people who just weren't in this [long‐term follow‐up care] system, there's nothing. […] I think these places are worth it for all current and former patients. It's that progress is being made, but those older survivors who simply aren't involved in this progress simply can't benefit from it, they are simply forgotten*. ‐ Female survivor, 16–25 years since end of treatment, late effects (Interview #21F)

## 4 DISCUSSION

In this qualitative study, we found that even long after the end of treatment childhood cancer survivors have many unmet needs. Survivors reported lacking psychosocial resources and support in long‐term follow‐up care and expressed a strong need for centralized long‐term follow‐up care centers. In recent years, four interdisciplinary long‐term follow‐up clinics have opened in Switzerland with one centralized clinic for long‐term survivors who were lost to long‐term follow‐up.^12^ To meet the needs of this growing population, long‐term follow‐up care for all Swiss childhood cancer survivors needs to be optimized.^8^ ^,^ ^12^ ^,^ ^14^

Despite standards of care recommending provision of psychosocial support for childhood cancer survivors also in long‐term follow‐up care,^1^ ^,^ ^30^ our findings show that such support is rarely provided. This could be interpreted twofold. First, current long‐term follow‐up might fail to identify and address survivors' needs for supportive psychosocial care.^31^ This is in contrast to accounts from specialists and generalists who reported to examine psychological late effects in all patients or at least if necessary.^32^ However, a recent study on HCPs' opinions on psychological screening in survivors' follow‐up care has shown that assessment of psychological late effects and provision of psychosocial support are not standardized in Swiss practice. HCPs pointed to limited resources and organizational barriers for offering psychosocial services as part of follow‐up care. Interestingly, HCPs did consider that psychological distress was sufficiently assessed in follow‐up care even if this was not done in a formal and standardized way.^33^ This stands in strong contrast to reports from survivors in our and other studies.^25^ ^,^ Future research should further tackle HCPs' perceptions of the benefits, barriers and practical issues with implementing psychosocial services in order to follow standards of practice and combat issues of undertreatment of psychosocial problems of childhood cancer survivors.^1^

Furthermore, childhood cancer survivors expressed a need for individualized information and care. In studies on long‐term follow‐up care or survivorship care plans, survivors have addressed similar needs and emphasized that personalized information should be provided.^18^ ^,^ ^34^ In addition, providing survivors with a long‐term follow‐up model that is individualized might increase their attendance and reduce additional costs to the health care system by providing care to those in need.^10^ For example, the “Personalized Cancer Survivorship Care Model” emphasizes putting the survivors' narrative at the center of care while considering their needs, values, and preferences. This model further includes risk‐stratification, in which survivors with a higher or moderate risk for late effects are followed‐up in a specialized long‐term follow‐up clinic. In contrast, for survivors with low risk the primary care physician is involved.^35^ ^,^ ^36^ Such a model allows for individualized and risk‐stratified long‐term follow‐up care, which includes an assessment of adverse physical and psychosocial effects.^14^ ^,^ ^36^ ^,^ ^37^ This is a successful example of long‐term follow‐up that recognizes the individuals' risks of late effects and their (changing) needs and concerns.^27^

Furthermore, childhood cancer survivors voiced three concerns that support a need for further institutionalization and coordination of centralized and interdisciplinary long‐term follow‐up clinics. First, survivors reported a lack of interdisciplinary collaboration amongst HCPs from different disciplines. Second, survivors experienced (dis)stress from having many different long‐term follow‐up appointments on different days, locations and with different HCPs due to decentralized care. Third, survivors illustrated that current decentralized long‐term follow‐up requires the survivor or their social environment to undertake action in organizing care. The challenge of navigating these three concerns has been documented in the literature.^26^ ^,^ ^27^ ^,^ ^38^ Indeed, barriers such as lacking information, interpersonal relations and practical and logistic challenges have prevented childhood cancer survivors from attending long‐term follow‐up. As a result, these concerns might hinder attending follow‐up, where survivors only seek care when faced with a medical problem, which goes against the preventative purpose of long‐term follow‐up care.

### 4.1 Study limitations

One limitation might have been self‐selection since some childhood cancer survivors may have been more reluctant or unable to participate,^28^ for example, survivors with severe late effects. This might have contributed to the under‐ or overreporting of unmet needs. The recruitment through Childhood Cancer Switzerland may have further biased our sample. Furthermore, despite self‐reported unmet needs, childhood cancer survivors might have memory bias given their young age at diagnosis. They might have received psychosocial support at some stages, but forgotten about it, or support was offered to parents. The findings of our study may not be generalizable to countries with different healthcare systems. The strength of our study lies in the in‐depth analysis of adult survivors' experiences during their survivorship, which allows for a nuanced understanding and recommendations for evidence‐based care.

### 4.2 Clinical implications

To meet the needs of survivors it is essential to provide easy access to long‐term follow‐up care for all childhood cancer survivors. Two recommendations can be made on the basis of our findings. First, more centralized interdisciplinary clinics should be developed. Second, psychosocial services should be implemented as part of long‐term follow‐up care, which is in accordance to international recommendations.^1^

## 5 CONCLUSIONS

Our study showed that psychosocial support in long‐term follow‐up care played a crucial role in childhood cancer survivors' unmet needs and should be included in future survivorship care.^12^ In addition, survivors expressed a strong need for centralized interdisciplinary long‐term follow‐up clinics. Lifelong specialized survivorship care and ongoing late effects education for survivors will help to improve health and mental wellbeing.^39^ We, therefore, recommend to include psychosocial services in long‐term follow‐up together with the development of personalized and evidence‐based long‐term follow‐up for survivors, and especially for very long‐term survivors after childhood cancer.

## CONFLICT OF INTEREST

The authors have no conflicts of interest to disclose.

## Supporting information

Supplementary Material 1

## Data Availability

Original data analyzed during this study may be provided by Dr. Hendriks upon written request.
