# Supplementary material for: The unmet needs of childhood cancer survivors in long‐term follow‐up care: A qualitative study
Source: Psychooncology. 2020 Dec 18;30(4):485–92. doi: 10.1002/pon.5593 (PMC8048447; doi:10.1002/pon.5593)
Supplement: Supplementary file 1 — Supplementary Material 1 [file PON-30-485-s001.docx]

| **Medical history and general information** | |
| --- | --- |
| *General socio-demographic data (to be filled in again or taken from the questionnaire)*  Participants are invited to talk about the impact of cancer on the life course (positive and negative aspects).   - Verbal and non-verbal encouragement to continue with the story ('what happened then?', 'why do you remember this specific moment?')   Can you say something about whether or not cancer has impacted your life? e.g.:   - School - Work - Families - Friends - Relations   What does the notion of health mean to you? |  |

| **Support - Experiences** | |  |
| --- | --- | --- |
| Can you say something about your experience with support after childhood cancer?   1. What kinds of types of support have you experienced? e.g.    - personal support    - psycho-oncological support    - professional support 2. Can you say something about your past/current/future support needs? |  | |

| **Support - Needs and preferences** | |
| --- | --- |
| What kind of support would you have liked?   - During and after cancer? - What should have been offered? - In what form? - Where?   Which services could you have benefited most from?  What kind of support could you need today, many years after the cancer?  What support measures would be useful or helpful for most former childhood cancer patients?   - When should the support measures be offered? - In what form? |  |

| **Concluding questions** | |
| --- | --- |
| What would you recommend to others [fill in, see below] who have experienced a similar situation? Regarding support, what would you recommend   - Other survivors? - Parents and family members? - Society? - Healthcare system / school / work?   Where do you see yourself in the future?  Questions / additions?  *Thank you so much for participating in this interview.* |  |
